# Different driver gene mutations in patients with synchronous multiple primary lung cancers: a case report

**DOI:** 10.1186/s13019-020-01178-z

**Published:** 2020-07-29

**Authors:** Yong Yang, Xiaofeng Xie, Gening Jiang, Hongcheng Liu

**Affiliations:** 1 Department of Thoracic Surgery, Shanghai Pulmonary Hospital, Tongji University School of Medicine, No. 507 Zhengmin Road, Shanghai, 200433 China; 2 Department of Pathology, Shanghai Pulmonary Hospital, Tongji University School of Medicine, Shanghai, 200433 China

**Keywords:** Multiple primary lung cancer, EGFR, ALK, Case report

## Abstract

**Background:**

Routine clinical and pathological examinations usually cannot fully conclusively determine the relationship between different lesions of lung cancer. Detailed genetic analysis of tumor samples may supply important additional information and identify second primary lung cancers.

**Case presentation:**

In the present study, we report a case of synchronous multiple primary lung cancer (MPLC) composed of two distinct pathological subtypes with epidermal growth factor receptor (EGFR) gene mutations L858R of the acinar adenocarcinoma subtype and *EML4–ALK* rearrangement of the squamous cell carcinoma.

**Conclusion:**

The present report highlights the clinical importance of molecular cancer biomarkers detection to guide management decisions in MPLC cases.

## Introduction

Multiple primary lung cancer (MPLC) is defined as two or more primary lung cancers occurring in the same patient and can be classified into synchronous or metachronous multiple primary lung cancer based on the time of occurrence [1]. Numerous patients with multiple lung tumors are encountered, due to the availability of high-resolution CT scan and the increasing incidence of adenocarcinoma histology among non-small cell lung cancers. The most commonly reported histologic type of MPLC is adenocarcinoma, which is consistent with the clinical phenomenon [2]. The distinct pathogenesis of lung cancer has not been fully clarified, in spite of somatic or germline mutations are believed to drive the development of lung adenocarcinoma [3, 4]. On the contrary, there are few MPLC cases reported with primary adenocarcinoma and squamous cell carcinoma. Moreover, to our knowledge, the different lesions of MPLC with different driver gene mutations have not been reported. It is important to elucidate the relationship between various lesions to make the best treatment strategy. A better understanding of the molecular alterations present in different lesions may help to define this relationship. In the present study, we report the cases of two synchronous lung adenocarcinomas, composed of two distinct pathological subtypes harboring epidermal growth factor receptor (EGFR) gene mutation and echinoderm microtubule-associated protein-like 4-anaplastic lymphoma kinase (*EML4–ALK*) rearrangement.

## Case presentation

**Fig. 1:**
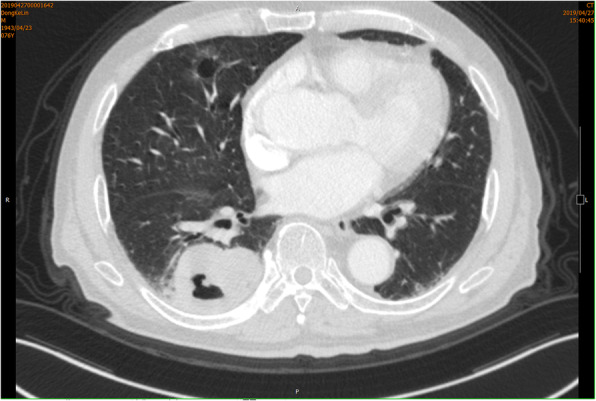
Chest computed tomography scan of the patient before operation. The right lung shows a 22 mm ground glass opacity with cavity in the middle lobe and a 61 mm solid mass with cavity and pleural indentation in lower lobe, with no hilar or mediastinal lymphadenopathy

**Fig. 2:**
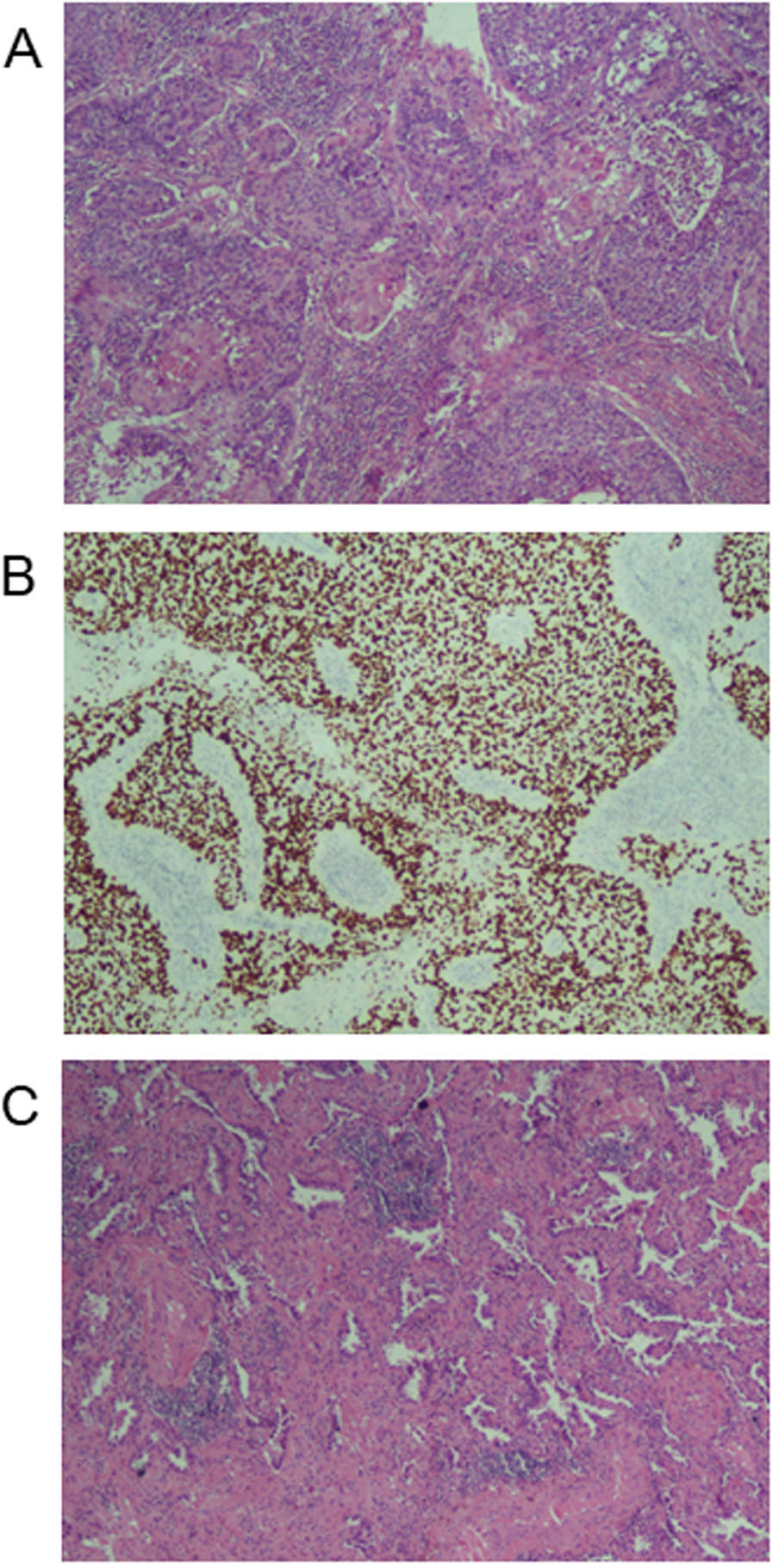
Pathology findings. **a**, keratinized squamous cell carcinoma of the lower lobe lesion. **b**, immunohistochemistry detection of P40 expression in squamous cell carcinoma. **c**, acinar predominant adenocarcinoma of the middle lobe

A 76-year-old male smoker with no family history was admitted to our hospital because of cough. Chest computed topography (CT) showed a 6 cm well-defined mass in the right lower lung, while another faint nodular lesion with a ground glass pattern and cavity was detected in the right middle lobe, together with emphysema and interstitial changes (Fig. 1). A positron emission tomography (PET) scan demonstrated negative findings for metastatic lesions, whereas brain MRI revealed a single lesion in the brain parenchyma that was considered as metastasis. Since there was only one distant metastasis and the cardiopulmonary function was normal, the patients received right middle and lower lobectomy by video-assisted thoracoscopic surgery (VATS). The pathology disclosed keratinized squamous cell carcinoma of the lower lobe lesion with visceral pleural elastic layer infiltration (PL1) and acinar predominant adenocarcinoma of the middle lobe (Fig. 2). Immunohistochemistry exhibited positive for CK5/6, P40 and P63, and negative for TTF-1 and NapsinA. Special staining of elastic fiber showed destruction of visceral pleura elastic fiber. Only group 11 lymph node was found with metastasis derived from squamous cell carcinoma. Specially, PD-L1 positive was > 60%, suggesting immunity therapy may be sensitive. Real-time polymerase chain reaction (PCR) analysis showed that the adenocarcinoma had an epidermal growth factor receptor (EGFR) mutation presenting as point mutation L858R, while the squamous cell carcinoma suffered from ALK fusion. The patient underwent radiotherapy for the brain and chemotherapy limited by the economic reason. There was also no detectable tumor growth in the half of year follow-up after the operation.

## Conclusion

Despite the low incidence of MPLC in non-small cell lung cancer (NSCLC) [1, 5], early detection and surgical treatment have been considered to be the best choice for outcomes [6]. Lung cancer shows the highest mortality rate among various cancers worldwide. In recent years, advances in CT scan screening led to the earlier discovery of lung cancer. Subsequently, a higher rate of multiple lung nodules has been detected on chest CT scans. MPLC suggests more than one solid lung mass identified in the same or different lobes or segments without treatment. However, most MPLC cases were found to have the same pathological type. In our case, we performed right middle and lower lobectomy for the patients. The EGFR gene mutation and *ALK-EML4* rearrangement in the lung adenocarcinoma and squamous cell carcinoma encouraged us to explore the tumor origin, and both tumors presented as treatment-naïve mutants occurring synchronously.

ALK rearrangement is one of the crucial molecular alterations in NSCLC, especially in adenocarcinoma with the incidence between 2 and 13% [7]. However, ALK rearrangement was detected with a frequency of lower than 1% in squamous cell carcinoma, thus was not routinely received molecular testing [8–10]. In this report, we observed one lesion of the patient harboring ALK rearrangement. Wang reported that in addition to having a fusion rate lower than that in adenocarcinoma, squamous cell carcinoma may also have ALK gene copy number gain and ALK gene mutation [11]. It suggested that the ALK alternative in squamous cell carcinoma might to be more complicated than that in adenocarcinoma. The relationship between squamous cell carcinoma and ALK gene status (including rearrangement, mutation, and copy number gain) still needs to be elucidated. Therefore, ALK status should be separately described for adenocarcinoma and squamous cell carcinoma. Similarly to the uncertain clinical efficacy of EGFR-tyrosine kinase inhibitor in squamous cell carcinoma, it is also controversy whether ALK rearrangement squamous cell carcinoma patients could benefit from ALK inhibitor. Crizotinib exhibited high response rates in advanced patients with ALK-positive NSCLC, especially brain metastasis [12]. In spite of the remarkable responses have been observed by some researchers [13, 14], it is still controversy about the efficacy of ALK inhibitor in ALK-rearranged squamous cell carcinoma [9]. A recent study indicated that the RAS-RAF-MEK-ERK signaling pathway determines the ALK inhibitor response in ALK-positive lung cancer, but still needs further investigation [15]. In our case, the postoperative pathology revealed that the squamous cell carcinoma suffered from ALK fusion mutation. Together with the brain metastasis, the patient received brain radiotherapy after surgery.

EGFR mutations have also frequently been reported in MPLC patients, whereas complicated mutations (L858R/S768I, G719X/T790M, L858R/20ins, 19del/T790M, 19del/L858R, and 19del/20ins) were only found in female patients [16]. Our male patient was an unusual case of synchronous double primary NSCLC with EGFR L858R mutations in adenocarcinoma and *EML-4ALK* rearrangement in squamous cell carcinoma. Our case was confirmed as stage I for adenocarcinoma (T1aN0M0), and thus the EGFR-TKI was not indicated. There are two main findings. First, the ground glass opacity in the middle lobe was not metastasized from the huge mass in the right lower lobe. Treatment naïve adenocarcinoma and squamous cell carcinoma occurred together in the same patient was rare, highlighting the importance of radical surgical intervention to improve the prognosis. Second, to the best of our knowledge, ALK fusion and EGFR L858R mutations in separate squamous cell carcinoma and adenocarcinoma diagnosed at the same time has not been reported. It showed that various kinds of driver gene mutations can present in different lobes simultaneously even from the very beginning [16]. Complex mutations suggested that tumor heterogeneity can exist in different tumors at the same time.

## Data Availability

The datasets analyzed during the current study was available from the corresponding author on reasonable request.

## Abbreviations

MPLC: Multiple primary lung cancer
EGFR: Epidermal growth factor receptor
CT: Computed topography
PET: Positron emission tomography
PCR: Polymerase chain reaction
VATS: Video-assisted thoracoscopic surgery
NSCLC: Non-small cell lung cancer
*EML4–ALK*: Echinoderm microtubule-associated protein-like 4-anaplastic lymphoma kinase
